# Supplementary material for: Content-rich biological network constructed by mining PubMed abstracts
Source: BMC Bioinformatics. 2004 Oct 8;5:147. doi: 10.1186/1471-2105-5-147 (PMC528731; doi:10.1186/1471-2105-5-147)
Supplement: Additional File 2 — The original results of the above study (non-essential files are deleted to keep the file size under the limit set by BMC bioinformatics). [file 1471-2105-5-147-S2.bz2 › chilibotAdditionalFile2/dip05/265ID7997270E1066/html/MAPK1_ARAF1.html]

 


 **MAPK1** and **ARAF1** 
  
Found 67 abstracts in PubMed, retrieved 05.  
 

 What does Google say? 
 PDF only 
| .edu only 

---

**Interactive relationship** (e.g. stimulation, inhibition, etc)

**Non-interactive relationship** (e.g. studied together, co-existance, homology, etc.)

- These studies suggest that the ability of Ras and BCR ABL to activate NF kB involves an uncharacterized pathway which does not involve classic IKK activity and which suppresses the TNF induced IKK pathway through a Raf  [ **ARAF1** ]  MEK Erk  [ **MAPK1** ]  independent mechanism.  Ref: 12851413 J Biol Chem< MedlineTA>J Biol Chem,
- Gastrin activated transcription through a Raf  [ **ARAF1** ] , Mek and Erk  [ **MAPK1** ]  dependent but Ras independent pathway.  Ref: 12891554 Gastroenterology, 2003
